# Genome Sequence of a Hyperthermophilic Archaeon, *Thermococcus nautili* 30-1, That Produces Viral Vesicles

**DOI:** 10.1128/genomeA.00243-14

**Published:** 2014-03-27

**Authors:** Jacques Oberto, Marie Gaudin, Matteo Cossu, Aurore Gorlas, Alexeï Slesarev, Evelyne Marguet, Patrick Forterre

**Affiliations:** a Université Paris-Sud, CNRS, UMR8621, Institut de Génétique et Microbiologie, Orsay, France; b Fidelity Systems, Inc., Gaithersburg, Maryland, USA; c Institut Pasteur, Paris, France

## Abstract

*Thermococcus nautili* 30-1 (formerly *Thermococcus nautilus*), an anaerobic hyperthermophilic marine archaeon, was isolated in 1999 from a deep-sea hydrothermal vent during the Amistad campaign. Here, we present the complete sequence of *T. nautili*, which is able to produce membrane vesicles containing plasmid DNA. This property makes *T. nautili* a model organism to study horizontal gene transfer.

## GENOME ANNOUNCEMENT

*Thermococcus nautili* 30-1 (formerly *Thermococcus nautilus*) was isolated on the East Pacific ridge near a black smoker, 2,600 m below the ocean surface (1). *T. nautili* qualifies as a new species and the characterization of its physiology and metabolism has been reported (2).

*T. nautili* was grown anaerobically in ZB medium at 85°C (1), and genomic DNA was purified and sequenced using paired-end Illumina libraries to generate 16 × 10^6^ reads assembled with Velvet (3). Genome assembly was performed with Consed (4). The remaining gaps were filled using bacterial artificial chromosome (BAC) sequencing with Thermofidelase and Fimers (5). Genes were annotated using FGENESB (Softberry). Additional functional annotations were obtained with Integrated Microbial Genomes (6). The chromosome of *T. nautili* is 1,976,356 bp long and harbors 2,288 genes. The noncoding genes comprise 4 rRNA genes (23S, BD01_1261, and 16S, BD01_1259), with a duplication of the 5S rRNA gene (BD01_1590 and BD01_2049), and 46 tRNA genes. The chromosomal origin of replication was identified with FITBAR (7) using published origin recognition complex (ORC) sequences (8). It is positioned upstream of the CDC6 gene (BD01_1824). A similar approach located the replication termination region at coordinate 772,784 using archaeal *dif* sites (9).

Several strains of *Thermococcus* contain either one or two plasmids (10–13). Our group previously sequenced and characterized two plasmids of 3.6 and 13 kb from *T. nautili* that were named pTN1 and pTN2, respectively (14–16). The rolling-circle plasmid pTN1 was used to construct the first shuttle vector able to replicate in both *Escherichia coli* and the model organism *Thermococcus kodakaraensis* (17) and contributed to the development of *T. kodakaraensis* as a host for genetics studies (18). Plasmid pTN2 encodes a new family of DNA primase formed by the fusion of protein domains homologous to the archaeo-eukaryotic primases PriS and PriL (19). DNA sequencing revealed the presence of an additional plasmid of 18 kb, pTN3 (16). This extrachromosomal element, related to the virus-like element TKV4 from *T. kodakaraensis* (20), is found as a freely replicating plasmid and also integrated at a specific position on the chromosome into a tRNA^Leu^ gene (BD01_0018). The plasmid attachment site (*attP*) is located within the integrase-encoding gene TnaP3-13 (16). The *attP* and *attB* sites share an identical stretch of 41 nucleotides (nt), as observed previously in the case of the TKV4 integron of *T. kodakaraensis* (20). In the chromosome of *T. nautili*, 6 clustered regularly interspaced short palindromic repeat (CRISPR) loci were found using CRISPRFinder (21). Interestingly, two CRISPR spacers are directed against resident plasmid pTN3.

A major interest in the study of *T. nautili* is its ability to generate membrane vesicles containing plasmid DNA (22), including the virus-like genome of pTN3 (viral vesicles) (16). The discovery that these vesicles can transfer genetic information between related *Thermococcales* (23) provides an important tool for the study of horizontal gene transfer across species or even domains, from which genomic evolution models can be inferred. The genetic determinism of the biosynthesis of these viral vesicles is presently being investigated and relies primarily on the *T. nautili* genome sequence presented here.

### Nucleotide sequence accession number.

The *T. nautili* genome sequence has been deposited in the NCBI repository under the accession number CP007264.

## References

[1] Lepage E Marguet E Geslin C Matte-Tailliez O Zillig W Forterre P Tailliez P 2004 Molecular diversity of new *Thermococcales* isolates from a single area of hydrothermal deep-sea vents as revealed by randomly amplified polymorphic DNA fingerprinting and 16S rRNA gene sequence analysis. Appl. Environ. Microbiol. 70:1277–1286. 10.1128/AEM.70.3.1277-1286.2004 15006744 PMC368356

[2] Gorlas A Croce O Oberto J Gauliard E Forterre P Marguet E 20 2 2014 *Thermococcus nautili* sp. nov., a hyperthermophilic archaeon isolated from a hydrothermal deep sea vent (East Pacific Ridge). Int. J. Syst. Evol. Microbiol. 10.1099/ijs.0.060376-0 24556637

[3] Zerbino DR Birney E 2008 Velvet: algorithms for de novo short read assembly using de Bruijn graphs. Genome Res. 18:821–829. 10.1101/gr.074492.107 18349386 PMC2336801

[4] Gordon D Green P 2013 Consed: a graphical editor for next-generation sequencing. Bioinformatics 29:2936–2937. 10.1093/bioinformatics/btt515 23995391 PMC3810858

[5] Malykh A Malykh O Polushin N Kozyavkin S Slesarev A 2004 Finishing “working draft” BAC projects by directed sequencing with ThermoFidelase and Fimers. Methods Mol. Biol. 255:295–308. 10.1385/1-59259-752-1:295 15020833

[6] Markowitz VM Mavromatis K Ivanova NN Chen IM Chu K Kyrpides NC 2009 IMG ER: a system for microbial genome annotation expert review and curation. Bioinformatics 25:2271–2278. 10.1093/bioinformatics/btp393 19561336

[7] Oberto J 2010 FITBAR: a web tool for the robust prediction of prokaryotic regulons. BMC Bioinformatics 11:554. 10.1186/1471-2105-11-554 21070640 PMC3098098

[8] Matsunaga F Glatigny A Mucchielli-Giorgi MH Agier N Delacroix H Marisa L Durosay P Ishino Y Aggerbeck L Forterre P 2007 Genomewide and biochemical analyses of DNA-binding activity of Cdc6/Orc1 and Mcm proteins in *Pyrococcus* sp. Nucleic Acids Res. 35:3214–3222. 10.1093/nar/gkm212 17452353 PMC1904270

[9] Cortez D Quevillon-Cheruel S Gribaldo S Desnoues N Sezonov G Forterre P Serre MC 2010 Evidence for a Xer/dif system for chromosome resolution in archaea. PLoS Genet. 6:e1001166. 10.1371/journal.pgen.1001166 20975945 PMC2958812

[10] Gonnet M Erauso G Prieur D Le Romancer M 2011 pAMT11, a novel plasmid isolated from a *Thermococcus* sp. strain closely related to the virus-like integrated element TKV1 of the *Thermococcus kodakaraensis* genome. Res. Microbiol. 162:132–143. 10.1016/j.resmic.2010.11.003 21144896

[11] Krupovic M Gonnet M Hania WB Forterre P Erauso G 2013 Insights into dynamics of mobile genetic elements in hyperthermophilic environments from five new *Thermococcus* plasmids. PLoS One 8:e49044. 10.1371/journal.pone.0049044 23326305 PMC3543421

[12] Gorlas A Krupovic M Forterre P Geslin C 2013 Living side by side with a virus: characterization of two novel plasmids from *Thermococcus prieurii*, a host for the spindle-shaped virus TPV1. Appl. Environ. Microbiol. 79:3822–3828. 10.1128/AEM.00525-13 23584787 PMC3675944

[13] Vannier P Marteinsson VT Fridjonsson OH Oger P Jebbar M 2011 Complete genome sequence of the hyperthermophilic, piezophilic, heterotrophic, and carboxydotrophic archaeon *Thermococcus barophilus* MP. J. Bacteriol. 193:1481–1482. 10.1128/JB.01490-10 21217005 PMC3067617

[14] Soler N Justome A Quevillon-Cheruel S Lorieux F Le Cam E Marguet E Forterre P 2007 The rolling-circle plasmid pTN1 from the hyperthermophilic archaeon *Thermococcus nautilus*. Mol. Microbiol. 66:357–370. 10.1111/j.1365-2958.2007.05912.x 17784911

[15] Soler N Marguet E Cortez D Desnoues N Keller J van Tilbeurgh H Sezonov G Forterre P 2010 Two novel families of plasmids from hyperthermophilic archaea encoding new families of replication proteins. Nucleic Acids Res. 38:5088–5104. 10.1093/nar/gkq236 20403814 PMC2926602

[16] Gaudin M Krupovic M Marguet E Gauliard E Cvirkaite-Krupovic V Le Cam E Oberto J Forterre P 1 8 2013 Extracellular membrane vesicles harbouring viral genomes. Environ. Microbiol. 10.1111/1462-2920.12235 24034793

[17] Santangelo TJ Cubonová L Reeve JN 2008 Shuttle vector expression in *Thermococcus kodakaraensis*: contributions of cis elements to protein synthesis in a hyperthermophilic archaeon. Appl. Environ. Microbiol. 74:3099–3104. 10.1128/AEM.00305-08 18378640 PMC2394913

[18] Farkas JA Picking JW Santangelo TJ 2013 Genetic techniques for the archaea. Annu. Rev. Genet. 47:539–561. 10.1146/annurev-genet-111212-133225 24050175

[19] Gill S Krupovic M Desnoues N Beguin P Sezonov G Forterre P 20 1 2014 A highly divergent archaeo-eukaryotic primase from the *Thermococcus nautilus* plasmid, pTN2. Nucleic Acids Res. 10.1093/nar/gkt1385 PMC3973330 24445805

[20] Krupovic M Bamford DH 2008 Archaeal proviruses TKV4 and MVV extend the PRD1-adenovirus lineage to the phylum *Euryarchaeota*. Virology 375:292–300. 10.1016/j.virol.2008.01.043 18308362

[21] Grissa I Vergnaud G Pourcel C 2007 CRISPRFinder: a web tool to identify clustered regularly interspaced short palindromic repeats. Nucleic Acids Res. 35:W52–W57. 10.1093/nar/gkm360 17537822 PMC1933234

[22] Soler N Gaudin M Marguet E Forterre P 2011 Plasmids, viruses and virus-like membrane vesicles from *Thermococcales*. Biochem. Soc. Trans. 39:36–44. 10.1042/BST0390036 21265744

[23] Gaudin M Gauliard E Schouten S Houel-Renault L Lenormand P Marguet E Forterre P 2013 Hyperthermophilic archaea produce membrane vesicles that can transfer DNA. Environ. Microbiol. Rep. 5:109–116. 10.1111/j.1758-2229.2012.00348.x 23757139

